# “See One, Sim One, Do One”- A National Pre-Internship Boot-Camp to Ensure a Safer "Student to Doctor" Transition

**DOI:** 10.1371/journal.pone.0150122

**Published:** 2016-03-02

**Authors:** Sa'ar Minha, Daphna Shefet, Doron Sagi, Haim Berkenstadt, Amitai Ziv

**Affiliations:** 1 Cardiology Department, Assaf-Harofeh Medical Center. Zerifin, Israel; 2 Sackler School of Medicine, Tel-Aviv University. Ramat-Aviv, Israel; 3 Shalvata Mental Health Center, Hod HaSharon, Israel; 4 Chaim Sheba Medical Center, Tel Hashomer, Israel; 5 Israel Center for Medical Simulation (MSR), Tel-Hashomer, Israel; Virginia Commonwealth University, UNITED STATES

## Abstract

**Introduction:**

The transition for being a medical student to a full functioning intern is accompanied by considerable stress and sense of unpreparedness. Simulation based workshops were previously reported to be effective in improving the readiness of interns and residents to their daily needed skills but only few programs were implemented on a large scale.

**Methods:**

A nationally endorsed and mandated pre-internship simulation based workshop is reported. We hypothesized that this intervention will have a meaningful and sustained impact on trainees' perception of their readiness to internship with regard to patient safety and quality of care skills. Main outcome measure was the workshop’s contribution to professional training in general and to critical skills and error prevention in particular, as perceived by participants.

**Results:**

Between 2004 and 2011, 85 workshops were conducted for a total of 4,172 trainees. Eight-hundred and six of the 2,700 participants approached by e-mail, returned feedback evaluation forms, which were analyzed. Eighty five percent of trainees perceived the workshop as an essential component of their professional training, and 87% agreed it should be mandatory. These ratings peaked during internship and were generally sustained 3 years following the workshop. Contribution to emergency care skills was especially highly ranked (83%).

**Conclusion:**

Implementation of a mandatory, simulation-based, pre-internship workshop on a national scale made a significant perceived impact on interns and residents. The sustained impact should encourage adopting this approach to facilitate the student to doctor transition.

## Introduction

Internship is one of the critical transition points in medicine, when the observational apprentice becomes an active and accountable practitioner. This transition is accompanied by considerable stress, with most interns reporting a sense of unpreparedness to this demanding year[1, 2], and more importantly—challenges patient safety and quality care aspects. Numerous medical schools programs have acknowledged this transition point and devised different training programs. Simulation-based curriculum was often chosen for its increasingly recognized efficacy and for its superior ethical approach[3, 4], and had demonstrated benefit in several locally based studies[5–8]. Based on this experience, the Israeli National Internship Committee in collaboration with the Medical School Deans' Association and the Ministry of Health, have decided to approach the critical transition point of internship on a national level and initiate a national, pre-internship “boot-camp”.

We hypothesized that implementing a national educational intervention which sets common standards of critical skills and communication skills, while promoting both self-confidence and patient safety, will have a positive, sustained impact on the transition from student to doctor.

## Materials and Methods

During 2003, a simulation-based workshop for internship preparation was developed by an appointed multidisciplinary advisory board and the Israel Center for Medical Simulation (MSR). Following two successful pilot workshops, the National Internship Committee and the Ministry of Health set this workshop as mandatory prior to the internship year. The 5 days program (See S1B Appendix) addressed selected core competencies, including:

- **Resuscitation—**Basic Life Support and Advanced Life Support.
- **Key manual skills—**Performance of wound suture, insertion of urinary catheter and naso-gastric tubes, alongside handling central lines and troubleshooting respiratory ventilator.
- **Cognitive skills—**Safe prescription writing and medical calculations for proper drug administration.
- **Communication skills**—delivering bad news, approach to domestic violence, legal/ethical issues (medical record, informed consent, disclosing medical errors).
- **Integrated skills—**team work, complex patient transport and hand-off.

In order to promote "hands-on" experience over frontal teaching, the workshop was preceded by a mandatory customized "e-learning" training module, which reviewed essential protocols and procedures in accordance with the workshop’s materials.

The workshop took place at MSR, a national, multimodality medical simulation center[3]. Small groups (6–7 participants) rotated between supervised simulation stations of low-tech models (e.g. wound suturing models), high-tech computerized simulators (e.g. SimMan®, Laerdal Medical; Wappingers Falls, NY) and standardized patients, enacting a range of clinical scenarios. The advanced clinical and communication scenarios were recorded and later jointly viewed for constructive feedback, with special focus on patient safety aspects. All instructors underwent a structured "train-the-trainer" workshop prior to tutoring, focusing on proper simulation-based education and video-based debriefing techniques.

In order to establish the efficacy of the workshop on the transition between student and interns, data was collected from workshops’ graduates that were at different stages of their professional career at that time (pre-internship, internship and post-internship-including both resident and certified practitioners). This allowed a wide perspective for the workshop’s efficacy over time and professional stage.

Graduating trainees were e-mailed an evaluation form (See: S1A Appendix), appraising their perception of the workshop’s contribution to different professional aspects.

According to the policies of the Institutional Review Board at the Sheba Medical Center, (Israel) studies which are based on anonymous and voluntary questionnaires are exempted from applying for formal IRB approval. The present study was reviewed the local IRB and was exempted from formal submission. Participants did not provide consent to participate in the study. Those who decided to respond did it voluntarily and were notified that the data will be collected and analyzed for research purposes. The data received was completely anonymous without any identifying information. The questionnaire consisted of closed questions, rated on the Likert’s scale of 1 (workshop contributed to topic to a minor extent) to 4 (to a highly significant extent). For simplification purposes, answers rating 3–4 are grouped together and referred to as a generally-positive contribution.

Statistical analysis was completed by utilizing SPSS v.22 (IBM corp. Armonk, NY). Continuous variables are presented as means ± SD. A two sided chi square test was utilized for examining intergroup differences. A two-tailed p value<0.05 was considered significant.

## Results

**Fig 1:**
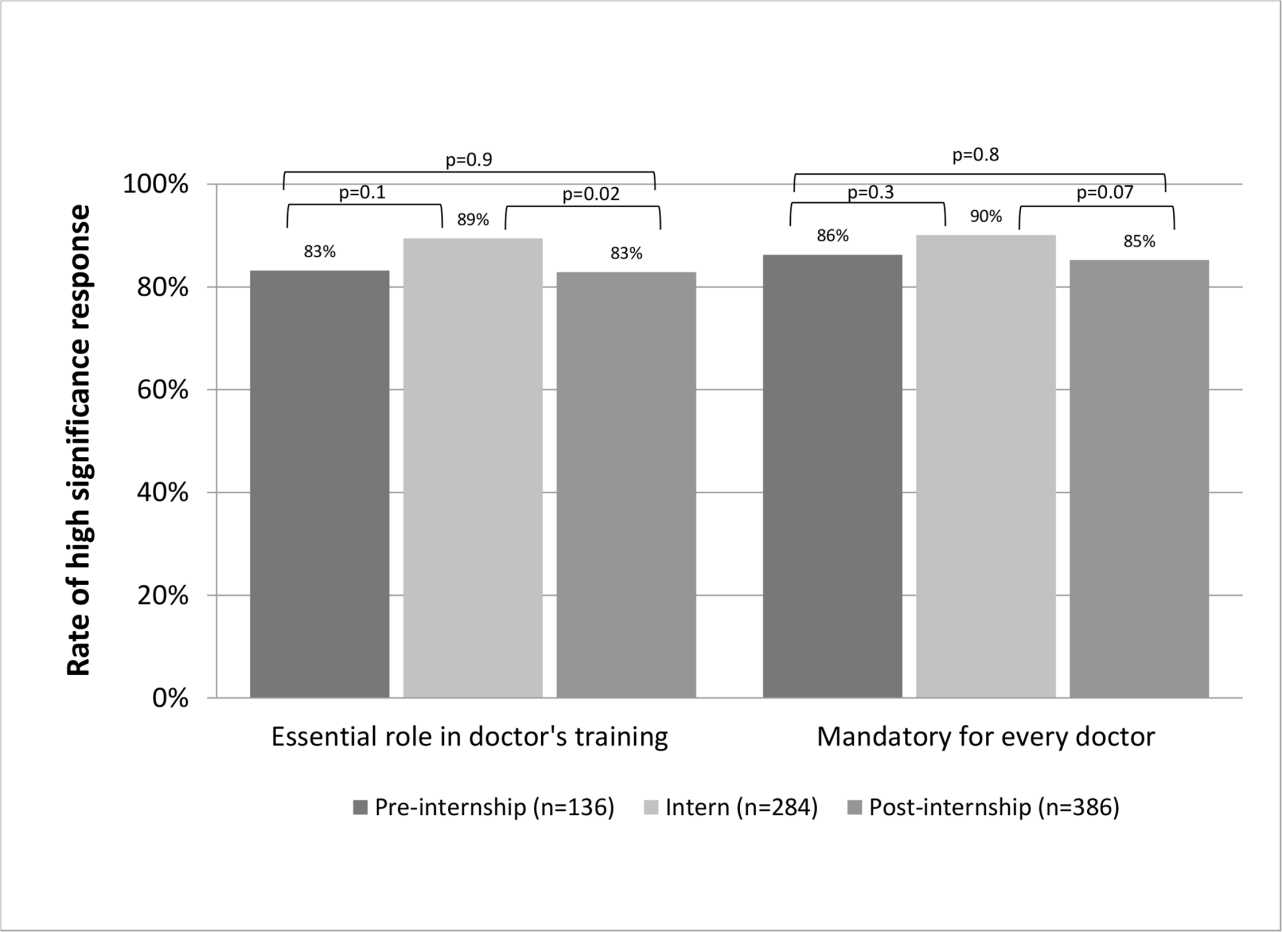
General perception of the workshop's role in the doctor training.

Between January 2004 to February 2011 this mandatory pre-internship boot-camp has trained 4,172 interns, through 85 workshops. E-mails were sent to approximately 2,700 trainees with known e-mail addresses. Over 500 fault addresses were identified. Eventually, 806 participants replied and the present analysis is based upon those responses (Fig 1). Fifty-seven percent of the responders were males. At the time of reply, nearly half of responders (48%) were either residents (mostly in the first half of residency) or certified practitioners, a third (36%) were interns and 7% had not yet begun internship. At most instances, pre-interns were scheduled to begin their internship within 1–2 months after workshop completion. Nine percent did not provide information on their training status.

**Fig 2:**
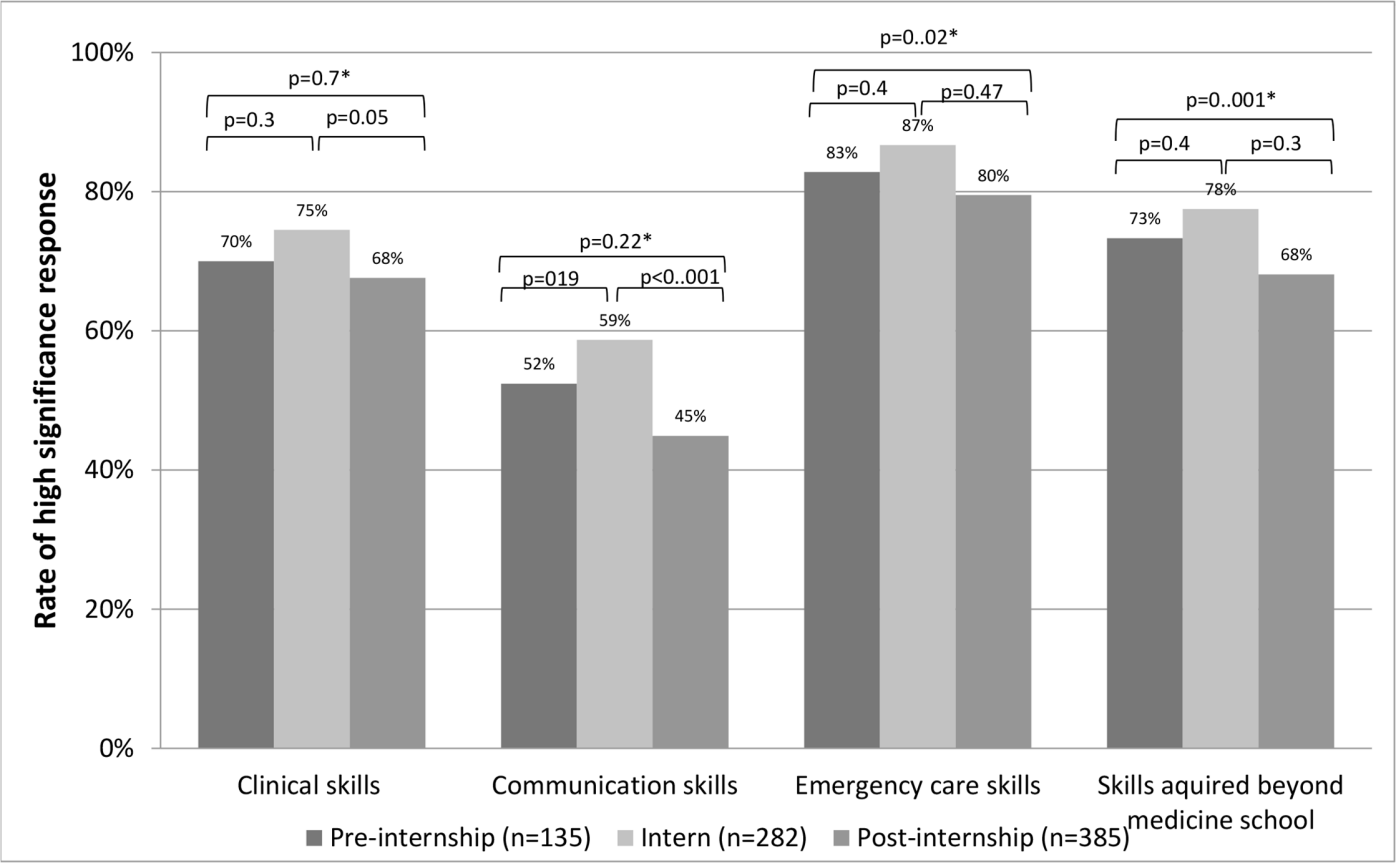
Perceived workshop's contribution to critical skills. *comparison of pre-internship vs. residents.

**Fig 3:**
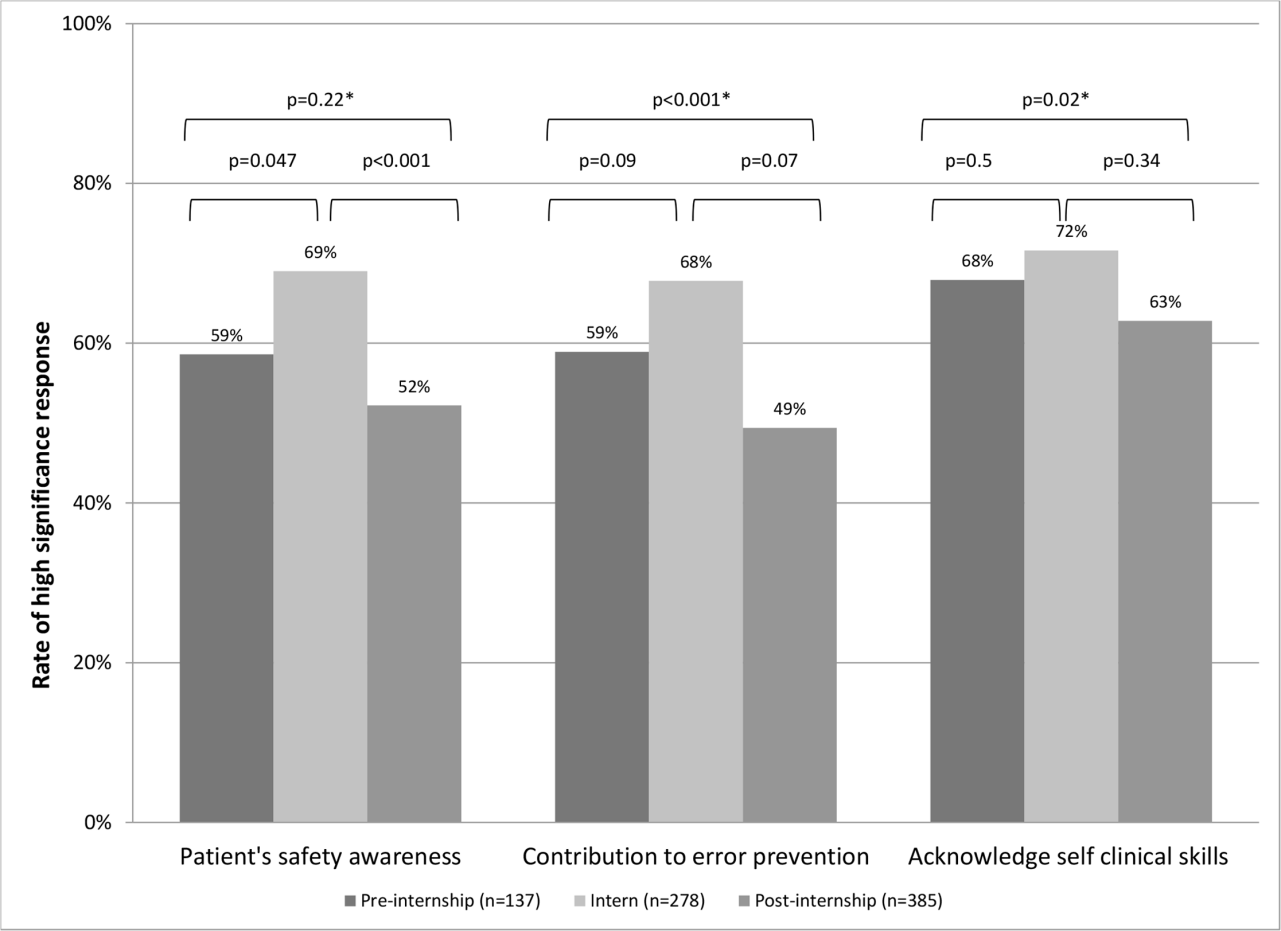
Perception of workshop’s contribution to error prevention and patient’s safety. *comparison of pre-internship vs. residents

Eighty five percent responded that the workshop is an essential component of their professional training (ratings 3–4) and 87% agreed that every doctor should participate in the workshop prior to internship. Although no significant difference was noted in subgroup comparison by career stage, interns had the highest positive rating for both questions. Positive perception of workshop’s contribution to both clinical and emergency care skills was rated by most responders (71 and 83%, respectably). This was also true for novel skills not previously acquired during formal medical school training (e.g. patient transport module, handling permanent catheters). Fifty-one percent of the responders rated positively the contribution of the workshop to communication skills. As presented in Fig 2, persistent positive perception of the workshop's contribution to the aforementioned skills was reported by trainees in different stages of their early career with highest ratings being reported by interns and some degree of decay in the contribution along the years. Similar to the clinical skills, most trainees reported that the workshop had positive contribution to their awareness to patients’ safety, error prevention and self-acknowledgment of clinical skills (59%, 57% and 67% respectively). When this is broken down to different professional stages (Fig 3), highest appreciation of the workshop’s role in error prevention and safety awareness is reported by interns. The positive perception of the workshop’s role in these metrics was generally maintained throughout and after residency.

## Discussion

This observational study of a national, pre-internship training workshop demonstrated persistent acknowledgment of skills needed for both safe and effective transition from student to doctor. To our knowledge, it is the first structured internship training program to be both nation-wide and obligatory.

Three main transitions exist in medical education: from non-clinical student to clinical student, from medical student to junior doctor and from specialist trainee to a medical specialist.[9] The second transition from being a medical student to an active and responsible intern dictates knowledge, critical skills and most importantly, integration between the two. Interns are expected to make clinical decisions and complete technical tasks without direct supervision (e.g. patient transport). This gap between the formal learning and actual expected performance is perceived as stressful by interns with over 90% of interns reporting to be not prepared for internship[1]. “Boot-camps” programs were thus developed to better prepare interns to their first year of practice with different emphasis on manual skills, decision making process and self-learning of protocols and procedures.[10–13] Most of these workshops utilized simulators and simulated patients in order to establish a monitored, safe environment promoting experience and confidence gaining without jeopardizing patients’ safety. Trainees reported of high satisfaction from these boot-camps immediately after graduating from the program.[14]

In Israel, the internship year is generic for all medical school graduates and this is followed by a residency in their field of choice. We thus opted to include skills needed for this generic internship year with the intention that those skills will further be utilized in the years to follow in different residency programs. The time elapsed between the program and its evaluation, found the participants at different stages of their careers—prior to internship, during internship and well into their residency. This viewpoint allowed establishing the long term effect of the acquired skills.

The rise in participants’ perceived contribution from pre-interns to interns, as seen across the board in all metrics, confirms the authenticity of scenarios and competencies selection. Despite the mild decay in perceived impact after the internship year, over 80% of residents still maintained the notion that the workshop was an essential part of their training and voted it mandatory, conveying the reassuring message is that all the aforementioned skills are persistently perceived as contributory even 3 years after workshop’s completion. Decay is expected and may be explained by both recall and memory bias (not affecting the overall reported relevancy of the workshop) and the fact that skills acquired by residents during the years of training may have affected the perceived contribution of skills acquired beforehand, during the workshop.

As unpreparedness for managing emergency care skills was previously reported by residents[15], it is not surprising that the highest perceived contribution of the workshop was recorded for those skills. While medical students are expected to memorize algorithms and perform “one-on-one” simulated training for basic life, higher integrated care tasks are expected from interns and these are not part of the formal training for medical school. Lowest contribution for future critical skills was recorded for communication skills. It has been reported that medical schools have improved in communication skills acquisition[16], and perhaps our results reflect this change; However, fear from emergency scenarios with potential morbidity and mortality may have exceeded that from potentially distressing human interactions, and if so—the comparison might have shadowed the latter’s value.

The model used to establish this integral training relies on three pillars- context authenticity, simulation fidelity, and timing. The context was chosen by experienced professional doctors but also from interviews of interns disclosing their “worse nightmare” during internship. Simulation fidelity was achieved by creating a hospital-like environment, including a hospital ward with nursing staff, family members and hi-tech simulators. All the scenarios were filmed and later viewed and discussed. Lastly, the interns were invited to participate in the workshop in close proximity to the first day of internship. It was expected that this “just-in-time” timing will have the highest effect on the trainees.

A good example of how those three pillars combine to promote patients’ safety and quality care, may be seen in a workshop’s transport module of a sick child accompanied by his father (an actor) crashing in the elevator. Intra-hospital transport, a common task for interns, is known for its hazardous potential to patient safety[17–19], and specific multi-disciplinary training was advocated by different authors in the past.[20, 21] Through this deliberately torturing scenario, of integrated resuscitation, communication, teamwork and decision-making skills, we practice critical skills and encourage interns to avoid taking tasks beyond their ability. Further, by allowing trainees to err, coupled with open and transparent debriefing and the introduction of adverse event and near-miss reporting forms, an important patient safety message is conveyed, which recognizes the presence of errors in the medical reality and encourages the reporting of a medical error in the future.[17]. Awareness to patient safety and error preventions was well reflected by the results of this survey.

We assume that the emphasis on instructors’ selection and training had significant contribution to the way the workshop was perceived by trainees. As mentioned, all instructors underwent a structured "Train the Trainer" training program developed for this workshop at MSR, establishing a uniform, goal-oriented tutoring. Inclusion of young mentors (some at the end of their internship year) in the team allowed for a less intimidating environment[22].

The limitation of this trial, lack of more objective outcome measures, is almost inherent to such national-scale interventions. While this study successfully shows a perceived major impact on real life training, obtaining objective measures in an intervention of this scale is highly challenging: The complexity of clinical situations, numerous participants, dozens of variables in real-life scenarios and national distribution, all make data collection and assessment practically unattainable. Achievements such as the importance of national recognition of the internship as a transition point, and the joint endeavors to meet those challenges, do not reflect well in tree plots and graphs. We should remember that immeasurable does not equal invaluable. Second, we acknowledge the response rate of the participants (36.6% after censoring the fault e-mail addresses). Although a higher rate of response would have contributed to the robustness of the study’s results, this response rate is somewhat expected from a survey sent by e-mail to extremely busy physicians years after completion of a workshop. We believe that data collected from over 800 participants during different stages of their career regarding a nationally endorsed pre-internship workshop has significant educational merit.

## Conclusion

This mandatory, national pre-internship workshop formally marks the internship as a critical transition point, demanding appropriate educational resources. Results establish its role and sustained relevancy for critical skills attainment and patient-safety promotion.

## Supporting Information

S1 Appendix Post workshop questionnaire and Workshop Educational Plan. (DOCX)

S1 Dataset Original dataset including full dataset, and original figures. (XLSX)
