# Supplementary material for: “See One, Sim One, Do One”- A National Pre-Internship Boot-Camp to Ensure a Safer "Student to Doctor" Transition
Source: PLoS One. 2016 Mar 2;11(3):e0150122. doi: 10.1371/journal.pone.0150122 (PMC4774927; doi:10.1371/journal.pone.0150122)
Supplement: S1 Appendix — (DOCX) [file pone.0150122.s001.docx]

Appendix A: Post workshop questionnaire

Dear Doctor,

Upon completion of your medical school studies, and prior to your internship, you had participated in a 5 day workshop at MSR (Israel Center for Medical Simulation). As you now have a perspective of time and clinical experience, we would like to hear your view on the workshop, in order to improve it for future trainees. The questionnaire is anonymous. Results will be summarized and collectively processed. We do however request a few details regarding your educational and professional background before and after the workshop.

Please read the questions carefully, and answer them all.

Thank you for your cooperation,

National Internship Committee and Association of Deans of Faculties of Medicine in Israel/ Ministry of health – Medical Board / MSR - Israel Center for Medical Simulation.

Top of Form

The following is a series of questions relating to the contribution of the workshop to your training. Please mark your answers to each of the questions on the following scale: 1 - minor extent, 4 - highly significant extent.

| 4 | 3 | 2 | 1 |  | |  |  |  |
| --- | --- | --- | --- | --- | --- | --- | --- | --- |
|  | |  |  |  | To what extent did the workshop contribute to the essential clinical skills required (from you) during internship? | |  | |
|  | |  |  |  | To what extent did the workshop improved contribute to your communication skills with patients? | |  | |
|  | |  |  |  | To what extent did the workshop contribute to your clinical skills in handling emergency situations? | |  | |
|  | |  |  |  | To what extent did the workshop contribute to your skills beyond the skills you had acquired during medical school? | |  | |
|  | |  |  |  | To what extent did the workshop contribute to your awareness to patient safety? | |  | |
|  | |  |  |  | To what extent did the workshop contribute to actual prevention of errors / near-miss events? | |  | |
|  | |  |  |  | To what extent did the scenarios practiced reflect situations you had encountered later-on as a physician? | |  | |
|  | |  |  |  | To what extent did the workshop render you to acknowledge your true clinical skills at the entry of the internship? | |  | |
|  | |  |  |  | To what extent should the workshop be an essential component in a physician’s professional training? | |  | |
|  | |  |  |  | To what extent is entering the internship stressful to young interns? | |  | |
|  | |  |  |  | To what extent does the workshop contribute to stress-reduction upon entering the internship? | |  | |
|  | |  |  |  | To what extent would you recommend this workshop as mandatory to interns before entering the internship? | |  | |

Please express your general opinion on the workshop

_________________________________________________________

In retrospect, which of the workshop simulated training modules were especially contributing to you?

 __________________________________________________________

In retrospect, did you lack exercises or training topics in the workshop?

 __________________________________________________________

Bring an example of a professional behavior you had acquired in the workshop and implemented later on as a physician

 ______________________________________________________________________________________

I graduated in: Israel / overseas

Year of workshop attendance: ____________

 Hospital of internship: __________________

Today I am a resident / specialist in ____________; If you had chosen another profession, please specify

 Today I work mainly in a hospital / clinic / other:________

 Gender: male/female

Appendix B- Workshop educational plan

| Day 1 | Day 2 | Day 3 | Day 4/5 |
| --- | --- | --- | --- |
| Opening lecture | Ventilator operation and troubleshooting | Drug calculation | Communication skills workshop |
| Lecture- teamwork | Acute asthma | Wound suture workshop | Complex scenario 1- pediatric transport |
| Lecture- medicolegal issues  Lecture- medicolegal issues | ACLS scenario-1 | Catheter insertion and handling | Complex scenario 2- cardiology |
| Lecture-BLS and ALS overview | ACLS scenario-2 | Transport module | Complex scenario 3- cardiology |
| Adult BLS | ACLS scenario-3 | Pulmonary edema | BiPAP/CPAP operation |
| Pediatric BLS | ACLS scenario-4 | Review exercises |  |
| Defibrillator operation |  |  |  |

Lecture

Technical workshop utilizing models and “simple” simulators

Medico-legal and communication skills issues (utilizing simulated patients encounters)

Hands-on basic simulated environment

Hands-on advanced simulated environment
